# Empowerment through participation in community-based participatory research—effects of a physical activity promotion project among socially disadvantaged women

**DOI:** 10.3389/fpubh.2023.1205808

**Published:** 2023-07-19

**Authors:** Ulrike Röger-Offergeld, Eva Kurfer, Hans Peter Brandl-Bredenbeck

**Affiliations:** Institute of Sports Science, University of Augsburg, Augsburg, Germany

**Keywords:** photovoice, focus group interviews, grounded theory, co-researchers, multiple empowerment levels

## Abstract

**Introduction:**

Community-based participatory research (CBPR) approaches are associated with a range of positive impacts on empowerment. However, only a few studies have investigated the influence of different modes of target group participation on empowerment. The present study examined the empowerment processes and outcomes of women in difficult life situations through their participation as co-researchers in the form of Photovoice in “Stark durch Bewegung” (English: “Strong through Movement”), a CBPR project for physical activity (PA) promotion. The extent to which women’s participation as co-researchers leads to empowerment was compared with other forms of participation.

**Methods:**

The Photovoice approach consisted of three components: (1) photo task, (2) focus group interviews, and (3) exhibition of photos. It was then extended through participant observation. A total of 18 women took part in Photovoice. They took photos, were involved in four focus groups, helped in the analysis of data, and supported their exhibition. Two additional short focus group interviews in which four more women participated were conducted at the end of the project. The interview guideline was based on the SHOWeD questions proposed within the framework of Photovoice and enriched with various other questions (e.g., self-efficacy, social contacts, and community involvement). The data were analyzed based on a grounded theory approach.

**Results:**

“Stark durch Bewegung” contributed to women’s empowerment in several ways. By participating in the project’s PA programs, the women reported numerous empowerment effects, such as improved self-efficacy, perceived competencies like swimming and language skills, and social networks. By participating as co-researchers, they perceived empowering processes on organizational and community levels that are comparable with other forms of participation (e.g., participation in a cooperative planning group) but also differ from them at relevant points (e.g., encouraging them to reflect on their own PA behaviors). The willingness to get involved in Photovoice was estimated to be significantly higher than in other possible forms of participation.

**Conclusion:**

Our findings support the notion that health promotion interventions with marginalized groups can contribute to their empowerment on multiple levels when participants become equal partners in the CBPR project. Involving women as co-researchers has advantages over other forms of participation in terms of their empowerment.

## 1. Introduction

The existence of social inequalities remains a central challenge in the field of prevention and health promotion. Health-related inequalities negatively affecting socially disadvantaged groups can be observed in all OECD and EU countries (1). Social disparities also exist in participation in PA and PA promotion, which is an important area of prevention and health promotion (2–4). This is especially true for women of low socioeconomic status (SES) and their leisure-time PAs (5, 6). Given the increased incidence of non-communicable diseases (e.g., cardiovascular disease, type II diabetes mellitus) in this target group (1), women with low SES could particularly benefit from the health-promoting effects of PA. Unfavorable conditions such as living in a deprived neighborhood, multiple burdens as a single parent, unemployment, and poverty often lead to relatively poor health and physical inactivity (7).

Tailored PA programs should address factors that encourage and help women with low SES to become physically active, including the high costs of PA programs, lack of childcare, and being too far from home (8). In the past, public health interventions were often conceptualized as top-down concepts mainly driven by professionals, and thus they often missed the needs of target groups. In recent years, however, participatory approaches to co-creation have become a central component of public health interventions and health promotion research (9, 10). Community-based participatory research (CBPR) approaches aspire to equally involve the target group, stakeholders, and researchers to combine “knowledge and action for social change to improve community health and eliminate health disparities” (11). They are based on several principles, such as participation and empowerment of participants, acquiring health literacy, building on strengths and resources within the community, genuine partnership, co-learning, capacity building among all partners, applying findings to benefit all partners, dissemination of results, and long-term partnership commitments (12, 13). CBPR focuses on and addresses the factors identified that hinder women from the target group from engaging in PA.

In CBPR, the extent of participation, as a key element in these concepts, is further described with different continuum models (14, 15). For example, Balazs and Morello-Frosch’s (15) continuum ranges from community members being research subjects to becoming research partners. In all models, a true partnership among target group members, stakeholders, and researchers is a crucial element and ideally relates to all phases of the research process. In this context, *co-creation* is an umbrella term to describe stakeholder engagement in different research and program process phases. It includes various modes of participation, such as co-planning, co-implementation, and co-research (9). Achieving a balance between research and action toward health equity is the gold standard (15). However, some authors question whether community participation in CBPR is necessarily a means to empower communities (16). Significant differences in stakeholder engagement in CBPR projects, ranging from full participation to no participation, were identified (17). Participatory research demands a great willingness and readiness from the participants to expose themselves to their own opinions, experiences, and personal views of the situation and to share power with the participants (18).

*Photovoice* is a CBPR method in which people are invited to participate as co-researchers and document their lives, take responsibility for how they want to present themselves, and describe their situation and living environment with accessible and easy-to-handle means (e.g., mobile phone). The theoretical framework of *Photovoice* derives from the principles of documentary photography, feminist theory, and the empowerment approach according to Paolo Freire (19, 20). The photos taken by members of the target group and brought into the research process challenge the researchers to look at the world through the same lens as the photographer (21). Furthermore, the method allows people to engage in dialogue with other stakeholders (e.g., policymakers) and advocates for change in their communities. It compensates for the existing language difficulties of target groups and supports a participatory empowerment process (22–24). The main difference between *Photovoice* and other photo-based research approaches, also known as *photo-elicitation* or *photo-interviewing* (25), lies in (a) who takes the photos, (b) the intention to use the photos to initiate actual change, and (c) the active participation of the interviewees in possibly all phases of the research process. Since its introduction through Wang and Burris (23), *Photovoice* has been used in health promotion to explore the views of different vulnerable groups. These include, for example, children and youth (24), people with disabilities (26), homeless people (27), residents of nursing homes (28), and women in specific life situations such as low-income, single-parent, immigrant backgrounds, and so forth (29, 30). In promoting PA, *Photovoice* was mainly used with children and adolescents, people with disabilities, and older people to investigate their attitudes, opinions, and factors that may hinder or promote their PA. Regarding socially disadvantaged middle-aged women, few *Photovoice* studies on PA and PA promotion (31, 32) concerning barriers, beliefs, and resources exist so far. Despite careful and in-depth analysis of literature databases, we could not find any studies in German-speaking countries.

One fundamental principle of CBPR is to promote processes by which participants gain more control over their lives (13, 33). Health promotion programs that focus on participation are associated with several positive elements of *empowerment* (34, 35). Different uses and meanings of empowerment can be found in the literature (36, 37). However, the view of Rappaport (1984), who defines empowerment as “a process: the mechanism by which people, organizations, and communities gain mastery over their lives” [(38) cited after (39), p. 43], is the one that is often shared in public health literature (39, 40). Different authors propose multiple levels of empowerment: individual, organizational, and community. From a theoretical perspective, it is also critical that a distinction is made between empowerment processes (“empowering”) and outcomes (“empowered”) so that the mechanisms through which empowerment supports and creates health are understood (39, 41, 42). At the same time, the latter differentiation leads to various understandings of the concept of empowerment (37). Zimmerman’s empowerment approach considers processes and outcomes on all three levels and is commonly used. In the following, we refer to these considerations and to “empowerment outcomes on the individual” level and “empowering processes on the organizational and community levels” (39). Our approach has been used in previous studies examining the empowerment of socially disadvantaged women through their participation in PA promotion (43, 44).

According to Zimmerman (39, 45), “empowerment outcomes at an individual level” may be classified into three components: intrapersonal, interpersonal, and behavioral. Intrapersonal refers to people’s self-image, expressed through ideas such as self-efficacy, perceived competence, and their motivation to be in control of their lives. Interpersonal relates to the understanding and feelings people have toward their community and related sociopolitical issues as well as to how people use analytic skills to influence their environment. Behavioral empowerment outcomes indicate the actual level of people’s involvement in formal (organizational) and informal (community) activities (e.g., informal groups and/or networks). At an “organizational level, empowering processes” offer opportunities for people to participate in organizational decision-making. By contrast, at a “community level,” activities that can lead to empowering processes include getting improved access to community resources, open government structures, and tolerance for diversity. However, given that empowerment by its very nature embraces the need to listen to the voice of the people, Zimmerman (39) highlights that the exact meaning of empowerment and the processes for bringing it about can only truly be understood in the context of its use. Nonetheless, the three components outlined are useful in classifying the types of activities that might be relevant.

A recent review showed that health promotion interventions positively impact the empowerment of different target groups (35). At the same time, it was concluded that most studies examine the effects at the level of individual empowerment while studies at the organizational and community empowerment levels are less common. Studies investigating the outcomes of different levels or modes of participation in CBPR projects are rare (17). Using the example of CBPR interventions to promote PA (1), it can be shown that perceived empowerment outcomes and processes depend on the mode of the participants’ participation (43, 44). Women who participated in the planning (co-planning) and implementation (co-implementation) of the project reported broader and multiple empowerment effects compared to those only involved in PA courses. However, program outcomes related to the participation of the target group as co-researchers have not yet been considered and compared.

The following primary research questions were addressed based on the abovementioned theoretical underpinnings.

• What empowerment processes and outcomes were perceived by women in difficult life situations through their participation in a CBPR project for PA promotion?

• Which empowerment processes and outcomes may be attributed to the participation of the women as co-researchers?

## 2. Materials and methods

### 2.1. CBPR project “Stark durch Bewegung”

“Stark durch Bewegung” (English: “Strong through Movement”) was a community-based participatory research project to promote PA among women in deprived and difficult life situations^1^ in a suburb of the city of Augsburg, Germany, which had high migration rates (67.7%). The community of health insurance companies in Bavaria funded the project from April 2019 to September 2022. The project used a participatory approach to involve the women actively in planning, implementing, and evaluating tailored PA programs and PA promotion activities. Other stakeholders involved in the project were Augsburg’s city council members, city council staff, and representatives of various community associations like sports clubs. At the beginning of the project, a project office (a position with coordination tasks) was set up in the Health Department of Augsburg. The office was responsible for coordinating the project activities and was financed by the health insurance companies. To monitor and evaluate the project, a position for a research assistant was also implemented at the university.

A significant challenge in participatory work with vulnerable groups is their equal engagement in the *co-creation process*, not least because of language issues and problems of power imbalances (9). Power must be shared to empower marginalized groups (46, 47). For this reason, a cooperative planning approach was chosen for *project planning* to involve women as *co-planners*. This approach ensures the effective involvement of all relevant stakeholder groups (target group, researchers, and other stakeholders), for example, by including a qualified moderator accepted by all participants and agreed-upon communication rules based on respect and equality. Furthermore, the ideas, priorities, and measures are formulated jointly throughout the planning process, with the moderator ensuring that everyone’s wishes are heard and equally included (47). Therefore, 6 target group women and 10 other stakeholders (city council members, city council staff, representatives of various community associations like sports clubs, exercise instructors, and staff members of the University of Augsburg) participated in the cooperative planning group, together with the researchers responsible for the project (*n* = 3). The cooperative planning group was led by a moderator, a staff member (research assistant) of the University of Augsburg, especially to assure equal engagement in the discussions of the participating target group. To support equality, the number and length of the individual participants’ speeches were likewise recorded and reported back to the moderator after each session. These details were considered in the next meeting to ensure the most equal involvement of all participating groups. Planning followed a standardized protocol (with the main issues of setting a planning goal, reviewing the ideas of the participants, prioritizing ideas, and developing actions) in seven planning group meetings.^2^ These meetings served to discuss the actions that need to be taken to improve the opportunities of women in difficult life situations to participate in PA.

Cooperative planning took place from October 2019 until June 2020, which was interrupted by the first COVID-19 lockdown in Germany. Commonly developed PA courses should have started in September 2020, but due to the second lockdown, the beginning of the programs was postponed to spring/summer 2021. Finally, the courses started online in May 2021 with 10 participants and then face-to-face in July 2021 with 41 participants in the first block of the programs (one block lasted 12 weeks). Owing to the long break, almost all women already recruited for the programs in September 2020 had dropped out and new participants had to be found. There were 75 and 111 women who participated in the second and third blocks, respectively (Table 1). Based on the wishes of the target group in the planning phase, four kinds of PA programs were realized in July 2021 after the Corona break: Moving in the Water, Walking to Running, Pilates & Dancing, and Self-defense/Fit-Bo. There was a high demand for the programs, especially for the water courses. Except for the course “Walking to Running,” which took place outdoors, each course was limited to a maximum of 15 participants by the insurance companies, following their prevention guidelines.

**Table 1:** All physical activity programs within the project period with numbers of participants.

Sports courses	Block 1 July 21–August 21	Block 2 October 21–February 22	Block 3 March 22–July 22
Water 1	7	12	13
Water 2	–	13	16
Water 3	–	–	18
Pilates 1	13	23	20
Pilates 1	–	–	18
Walking	9	12	10
Fit Bo	12	15	16
**Sum**	**41**	**75**	**111**

The following framework conditions highlighted as important by the women in the cooperative planning process were also implemented: availability of childcare, close to home, no cost, and PA programs for women only (safe space).

*During project implementation*, the city officials rejected the intended employment of the target group women on a mini-job basis to support the project office manager (co-implementation) in the Health Department of the city of Augsburg. Nevertheless, the women were still able to participate on a more informal basis, if they desired, in this phase: they helped with participant recruitment, assisted exercise instructors with participant lists and Corona testing, were involved as PA instructors, and helped to organize meetings besides training (breakfast together), among other activities. For most of these activities, they received monetary compensation.

Women used a *Photovoice* approach to serve as co-researchers (co-evaluation) in the project evaluation (22, 48). The *Photovoice* approach was based on a standardized process. Participating women took the photos (Step 1), interpreted the photos together in focus group discussions and a separate evaluation session (Step 2), and shared the themes identified with policymakers and other stakeholders by organizing and holding an exhibition together with the researcher (Step 3) (22, 49).

We used the *Photovoice* approach and participant observation to collect data based on the above considerations.

### 2.2. Data collection

A three-component strategy was applied to implement *Photovoice* for data collection, which was extended by participant observation (fourth component).

(1) The *first component* was to give participating women a photo assignment (“Take photos of your everyday life around the theme of PA and sports”) to enable them to collect data relevant to them. The researcher invited all women joining the PA courses in person and via WhatsApp to participate. The *Photovoice* participants were asked to use a smartphone camera to capture about 8–10 photos within one week. They could send them to the researchers via WhatsApp or Wetransfer.com. All the women chose WhatsApp since it is their daily communication tool.
(2) During subsequent focus group discussions (*second component*), the target group members had the opportunity to collectively interpret their photos, examine their personal views compared with other participants, and discuss them in critical dialogue. Using the photos as stimuli, the focus group discussions were also based on an interview guideline to enable a similar process across the four different focus groups. The guideline consisted of three sections: (a) opening phase, (b) questions about the photos, and (c) expanding questions. In the *opening phase*, the participants were welcomed, and the procedure and general conditions (e.g., recording, anonymity) of the focus group discussion were explained. The *questions about the photos* were based on the SHOWeD guide developed by Wang and Burris (49) and adapted to our context. The opening question was formulated openly to initiate a free conversation without raising expectations. The first question was: How was the photo task for you? Moreover, this phase was based on the following questions: What do you see here? What is really happening here? How does this photo relate to your lives? What is essential for you in this photo? What meaning does the picture have for you? The expanding questions were focused on the following areas: purpose and special features of the PA courses, changes in PA behavior, general changes (e.g., nutrition, self-efficacy, social contacts), cooperation with partners (exercise instructors, city of Augsburg, University of Augsburg), wishes, and suggestions for improvement. With the women’s consent, each interview was audiotaped and wholly transcribed. Data analysis was based on a grounded theory approach, and all group interviews were conducted by sequence analysis (see data analysis section below). After a first evaluation by the researchers, the women who participated in the group discussion were asked to join a separate evaluation meeting to discuss, improve, and expand the preliminary results. They all showed up, and another seven women joined the group to share their views and opinions on the project.
(3) At an exhibition, selected photos and interview quotes from the group discussions and a video produced especially for the occasion were presented as a *third component*. All women participating in the group interview were asked if they would like to show their photos and interview quotes at the exhibition. To ask them, the researcher joined all PA courses for two weeks to reach all participants, all of whom (*n* = 18) agreed. For the video, again, all women in the project (75) were asked by the researchers during the PA courses and via WhatsApp, in German, Turkish, Persian, and Hebraic languages, if they wanted to be a part of it. Fifteen of the women agreed to participate. The video showed 1–4-min sequences from the participating women telling who they are and what the project means to them. The video recordings were taken shortly after the PA courses, so no extra effort was required from the women. The exhibition aimed to sensitize relevant decision-makers in Augsburg to the women’s situation and share the project’s benefits with them and the public. Additionally, it should show that the project not only opens an avenue to improve mental and physical health but can also create cultural openness and social integration, promote democracy, start an empowerment process, and open an opportunity for dialogue. All women participating in the PA courses were invited by the researchers at the courses and via WhatsApp to contribute actively to the design and implementation of the exhibition. A total of 10 women followed the invitation, 8 of whom had already participated in the *Photovoice* study. The exhibition took place in a special showroom in the city center of Augsburg and lasted 12 days (July 12–23, 2022). At the opening ceremony of the exhibition, all women participating in the project, the exercise instructors, stakeholder groups, politicians from various parties in Augsburg, the Equal Opportunities Officer, interested citizens, and the local press were invited. About 60 people attended the event. After the exhibition, 11 women (7 participating in the *Photovoice* and 4 contributing to the exhibition) were interviewed in short focus group interviews. The researchers asked them, “How was the exhibition for you?” They were likewise given the chance to describe their thoughts about the exhibition and the project in general. The exhibition was meant to create publicity and support a sustainable implementation within the city council structures beyond the end of the project.
(4) Besides the group discussions, participant observation took place from October 2022 to July 2023 as a *fourth component*. To enable proper documentation, the researcher joined all courses at least twice a month, from course block 1 to course block 3. During the PA courses, these observations and conversations were logged by minutes, notes, and memos.

### 2.3. Sample

To reach the women for the **photo task** and **subsequent focus group interviews**, the researcher actively joined all PA courses within the first eight weeks to get to know the women and build trust. Owing to COVID-19 and an announced third lockdown in Germany, the researchers decided to use this mode of data collection at an early stage of the project in December 2021 and January 2022. Therefore, the women were recruited from block 2 of the programs (Table 2). For the photo task and subsequent focus group interviews, all women participating in the four PA courses in block 2 were asked. For each group discussion, 3–5 women attended, and the duration was between 40 and 70 min. Out of 75 women, 18 agreed to participate (Table 2), 17 of whom had an immigration background. Only 1 woman did not show up. Instead, 3 additional women came to the group discussions without informing the researcher beforehand; they said they were interested in the group discussion and wanted to take the opportunity to speak German and talk to other women from different countries. Given the long Corona break, almost all women who initially participated as co-planners in the planning phase had left the project. Since a co-implementation employing a target group of women to support the project office in the city of Augsburg could not be realized, the 18 women involved in *Photovoice* were primarily engaged in the project as co-researchers.

**Table 2:** Number of focus group interviews and participants in block 2 of the PA courses.

Group interview	Swimming	Walking/running	Pilates/Dancing	Fit Bo/Yoga
Number of participating women	5	5	3 (+2 listening women)	3
Nationality	5 from Turkey	2 from Turkey, 3 from Afghanistan	5 from Turkey	1 from Germany, 1 from Iran, 1 from Afghanistan

After the exhibition, 11 women (7 participating in *Photovoice* and 4 contributing to the exhibition) were interviewed in the form of **two short focus group interviews**. For the additional **participant observation**, the researcher actively joined all PA courses at least twice a month.

### 2.4. Data analysis

Reflexive grounded theory was conducted to analyze the data from all interviews (50). The characteristics of this theory are a circular-iterative approach and hermeneutic interpretation work. Reflective grounded theory emphasizes the importance of the researcher and the research interaction for knowledge formation.

In each phase of the research process, there was a constant interaction and analysis with the topic of empowerment, the research setting, the target group, and the two researchers themselves. After a first evaluation of the researcher (see first step below), all interviewed women were involved in the form of a group interpretation session with the researcher. They added details missing in the interviews, corrected the researcher’s interpretation when wrong, and reflected on their empowerment. Language barriers were challenging as two women could not read or write and most of them could not speak German very well. Again, because we had women with different mother languages, we decided not to have a translator. During further data analysis of the researcher (see second step below), the participating women were involved on a regular basis. The researchers wrote independent memos to focus on and elaborate the thoughts and reflexive attitude. The results were compared several times. All phases were accompanied by literature research to compare and reflect the findings.

For the **data analysis, open, axial**, and **selective coding** were conducted (50): In the **first step** for all group interviews, the starting sequences were **openly coded** line-by-line to analyze and compare as many small units as possible since no categories should exist at the beginning of the process. These first findings were written down in memos, discussed, and adapted. This step added conceptual codes and notes to a manageable number of categories. At this point, it was decided which phenomenon would be observed based on its frequent occurrence, namely, empowerment. The primary type of empowerment provided the key to understanding the fundamental problems and theoretically integrated the (partial) concept found and developed. The **second step** was **axial and selective coding**. The findings were systematically ordered, related to one another, and categorized by comparing them with similar and different cases to extend, validate, and consolidate the modeling. Categories were found, invented, constructed, and elaborated only during analysis. The two researchers worked partly independently of each other, and their results were repeatedly compared. This step was carried out until a theoretical saturation degree was reached. The aim was to formulate a theoretical model of limited range, which is presented in Section 3. An overview of the resulting category system is shown in Table 3.

**Table 3:** Overview of the category system.

Main category	Individual level	
**Subcategory**	**Intrapersonal level**	
Domains	Self-efficacy	• Confidence to be physically active regularly
		• Getting access to adequate physical activity offers
		• Positive impact on their daily lives
		• Confidence to take the role of the exercise instructor
		• Belief in understanding the photo task
		• Confidence in talking to responsible persons in the city
		• Confidence in being heard by city officials
	Domain-specific perceived control	• Facing their fear of water
		• Facing daily pain issues
		• Overcoming their fatigue/depression
	Perceived competences	• Letting go of the poolside
		• Learning how to swim
		• Improving swimming techniques
		• Enhancing nutrition skills
		• Improving language skills
**Subcategory**	**Interpersonal level**	
Domains	Critical awareness	• Awareness of the importance of the support of the group and the exercise instructors
		• Awareness of the importance of support from the husband and/or children
	Understanding causal agents	• Understanding causal agents in the community
**Subcategory**	**Behavioral level**	
Domains	Social Contacts and networks	• New social contacts and networks

Main category	Organizational level	
Domains	Assisting others	• Assisting instructors
		• Assisting project coordinators & researchers
	Taking on independent tasks	• Role of a exercise instructor
		• Role of a co-researcher
	Participation in decision-making	• Involved in the development of program contents throughout the program
	Initiating decision -making processes on their own	• Initiating swimming courses
		• Expressing their needs and wishes to city officials as a primary stimulus for project sustainability

Main category	Community level	
Domains	Access to communal resources	• Access to communal spaces like public indoor swimming pools, public gyms, and exhibition room
	Sense of community	• Cultural openness
		• Women stood up for other women
	Open government structures	• Speaking • At different events in the community (exhibition, project closing ceremony, etc.)

The following Results section concentrates on the interview quotes of the women. The presentation of the photo data would go beyond the publication’s scope and will be done elsewhere.

## 3. Results

The women reported different effects on all three empowerment levels defined in the introduction, namely, individual, organizational, and community.

### 3.1. Individual level

On an individual level, the interviewees/co-researchers mentioned various aspects of perceived empowerment which cover intrapersonal, interpersonal, and behavioral aspects. *Intrapersonal aspects* refer to how people think about themselves, *interpersonal aspects* relate to people’s understanding of their environment and community, and *behavioral aspects* concern people’s involvement in the community.

#### 3.1.1. Intrapersonal component

##### 3.1.1.1. Self-efficacy

All women reported changes in **self-efficacy** through participating in the project. Self-efficacy refers to **confidence in one’s ability to perform a particular behavior successfully or achieve a desired goal**.

**Regarding** PA **behavior, almost all women reported “being more confident in being physically active regularly”, and—very importantly—they attribute their increased activity level to their initiative**.

I move more. Otherwise, I would be at home. I would just sit in front of the TV and not do much. I'm the type who doesn't go out alone. I have to force myself. This project is excellent for me; I only have Monday and Tuesday off. Wednesday, I run. I am happy about that. In our country, they say, “I pull myself with the rope on this day.” Wednesday is running, Thursday is swimming, and today is Pilates (Pilates, Woman A).

Only a few women were regularly physically active before joining the project. For example, one swim class participant was already jogging alone every day. She taught herself to run longer distances and, over the course of the project, developed the “confidence to take on the role of a PA instructor” in the program (Walking to Running) and coach the other women.

I quit smoking five years ago. Then I started to do sports because I had gained too much weight; I had to start. It was such a short distance in the beginning, of course. First, 1 kilometer then back again. But now I can do it every day. I feel bad for not doing it (Swimming, Woman B).

**Accessing adequate PA courses** was an essential precondition for the women to participate regularly; the courses needed to be appropriate to their lives and settings. During the planning phase of the project, the women emphasized the importance of the courses being close to home and that women-only courses provided a safe space. In the interviews, they expressed excitement about getting such programs and thus achieving their goals. This was especially true for the participating Muslim women because, so far, there were no programs for women only and free of charge in the city of Augsburg.

But with us, there are limits. For example, we are not allowed to mingle with strangers. Women are okay, but not the men. But since we didn't have the opportunity for a long time, we are really happy that now there is the opportunity, and we want to continue. For us, it's the first time we are getting something like this (Walking to Running, Woman B).

For non-Muslim women, these above conditions were essential aspects of their participation as well.

That's why I don't need the gym, with all the musclemen sometimes in there or gawping at you. So I like it more that I can train by myself or with friends (FitBo, Woman A).

Some women said that the perceived effects of regular PA **positively impact their daily lives**. They experienced their otherwise rather meaningless everyday life—as they describe it themselves—with new meaning and structure. This was especially true for women whose children had just left home. Women with young children also reported effects such as enjoying the company of other women and doing something for themselves and not the children, housework, etc.

It is so with me; I now always have programs, weekly programs, but now they are even more programmed in my life, the days and weeks. That's quite great. I have to; this day I go to sports and so on. That's neat, and sports are even more a part of my life (Walking to Running, Woman B).

The focus group discussions revealed differences among the women regarding their **belief in understanding the photo task**. Some women seemed and believed to have understood the photo task and were the first to speak in the group interviews. They were confident about the starting question of the interview, their photos, and how they got along with the task. These women wanted to share their pictures and experiences. This group mainly comprised women with good German language skills and sporting backgrounds.

With photos? Yes, I took pictures when I was running and in the kitchen. And then I (photographed) my prayer rug. Because I pray three times a day, I said to myself, “I'll photograph it” (Walking to Running, Woman A).

I was jogging, so I took the first one (Walking to Running, Woman B).

Others seemed quite unsure or needed help understanding the photo task.

That is, (doing) it (photos) yourself is wrong, that is so. In the beginning, I also did not understand (the photo task). But (photograph) everyday life, you wrote afterwards (Walking to Running, Woman B).

I didn’t understand at first, either. Then I understood (Walking to Running, Woman E).

The latter women took pictures of them doing housework, cooking, of home-cooked food, their children and grandchildren, and meeting with friends. Hence, they were initially unsure whether to show their photos to the others, as the photo task involved photographing one’s everyday life around PA and sport. Throughout the interviews, they reflected on their everyday life through PA, sports, and potential barriers to participation based on the photos together with the other women. As the interviews progressed, the women received confirmation of their pictures from the group and became confident. They even agreed to show the photos at the exhibition.

It was also an opening of the personality in such a way that we showed ourselves personally, familiarly, the way we live, or what we do, not only in writing but also through pictures. These are already personal things, what you want to show or what not to. One wanted to help the other women so they could participate. There was such a desire from the women, but there was also a demand. You show that, and then you somehow open doors and gates (Interview after Exhibition, Woman B).

During the presentation of their photos at the exhibition and their participation in the opening ceremony together with political decision-makers and other city stakeholders, a few women were strengthened because they could **talk to persons of authority in the town** and **were heard by them**.

No, it was already good; it was a shaking up that we could say that we were also here. We are invisible in the city because we are not loud, but we are there. Society is like that; you can’t just look away. You can´t ignore the refugees or the Ukrainians, or, I don’t know, foreigners; they are just there in the city. You can only do something together. Side by side, it would only work for a short time. There would be big problems. And you can only be noncommittal in such groups, get to know each other, and accept and respect the others (Interview after Exhibition, Woman A).

That we are taken notice of by the city, that we are also there (Swimming, Woman C).

##### 3.1.1.2. Domain-specific perceived control

The women also reported domain-specific control effects related to perceived pain (e.g., back pain), overcoming fatigue and anxiety from water, and in general. Perceived control is defined as the belief in one’s ability to control situations or events (45).

Several Turkish women talked about their **fear of water**, often triggered by negative experiences with water/swimming in the past, as most of them did not know how to swim. This aspect seemed very stressful for all women concerned, and it seemed important for them to overcome this fear. They face it, but not alone, without anyone watching, but in the presence of the group and the exercise instructor.

Then I didn't go swimming for a year. I didn't go to the sea anymore either. Then my daughter forced me; you have to overcome your fear somehow. Why don't you go to the sea again? And so slowly, I did it again, but I always get this panic when I'm in deep water. I could swim, but I don't know where this panic comes from. It has always accompanied me (Swimming, Woman D).

One of the interviewees said she called all her family members, friends, and the researcher to tell them that she had overcome her fear of the water and could swim now. After overcoming their fears, some women even switched roles and helped other women with fears in the swimming courses. They were proud of themselves, gained self-esteem, and showed this openly to others. This outcome was especially true for Turkish women, who usually go to the beach on vacation in Turkey during the summer.

Another critical issue for the women was **daily pain**, such as back pain, intervertebral disc problems, and Achilles problems. Participating in regular PA, especially swimming, helped them manage and relieve their pain. They even reported that their pain returned when they did not participate in the PA programs of the project for a week or two.

I also noticed my vertebrae and my neck; swimming helps a lot. I also noticed that. And when I'm in the water and feel my legs, I feel good, have no pain. Or if I stand, then I'm even fitter. But when I stopped, I noticed that I saw a pulling again (Interview after Exhibition Woman A).

Furthermore, many of the interviewed women stated that they were able to **overcome their fatigue and depression through regular PA**. They explained that they felt tired in their daily lives and experienced how PA helps them overcome this fatigue. They perceived that it enabled them to be positive and feel more energetic daily. The following quote shows the psychological importance of the project for many women. They regain control of their lives, at least for a few hours a week.

I have less stress. I think, “Ah, Monday is swimming. Good, a little exercise is good.” And Friday, I will wait until Friday. Friday is also good; I do Pilates. I refrain from thinking about so much wrong; always think good. It’s good for my brain, it’s good for everything and the body. And I have less back pain. Less pain in my legs too. Sport is always good. Stay healthy (Pilates, Woman C).

##### 3.1.1.3. Perceived competencies

The women perceived that they acquired different competencies through participation in the project. Some addressed that they learned **to let go of the poolside** while others **learned how to swim** or **improved their swimming techniques** by participating in the project’s swimming courses. One said she thought she knew how to swim already but found out at the beginning of the swimming courses that her technique was not good. Another woman said that learning the techniques and getting them explained in detail helped her by teaching her children how to swim, which she had not been able to do before.

Also, in the pool, I'm just on the edge of the pool, [Name of the exercise instructor] knows. She said, “Now let go.” But this fear is so big. *Interviewer*: But now you let go. *C*: Thank God, but it took a long time. For years (Swimming, Woman C).

Concerning the other PA offers of the project (e.g., Pilates and Walking to Running), the women indicated that they **felt fitter**. Specific motor competencies acquired were not articulated in their context. For them, walking means going for a walk together, talking with others in the fresh air on the riverbank about their daily lives, challenges, and questions about jobs, the German system, or educational topics.

Well, I also think that the condition is strengthened a bit if you train regularly. So, of course, the essential requirement is there, but if you then train regularly, then it tends to stay and doesn't fall off again, and then you have to build it up again ultimately. I have already noticed that (FitBo, Woman B).

The women likewise reported the acquisition of competencies related to **healthy eating**. Although it was not an explicit topic for the semi-structured group interview, they talked about their definition of healthy food, what they try to do to stay and eat healthy, and where they buy healthy products. Almost all women took a picture of their home-prepared meals, even though the photo task was to take pictures of everyday lives in terms of PA and sports. Some did not understand the photo task. This could be taken as a sign that nutrition is essential to them. For this reason, they frequently asked the exercise instructors about healthy food and how to lose weight.

I make so much dough stuff, börek, and stuff. Now I have fewer sweets in my kitchen. I try hard because I'm 50 years old, and my kids must learn about correct nutrition (Walking to Running, Woman B).

Most of the women with a migration background wanted and were able to improve their **language skills**. They have experienced that language skills are crucial to participation in foreign societies and to opening new opportunities. The reasons some had not previously learned German fluently were as follows: language courses were too expensive, they did not know where they could get the information, they did not have time, they were not sure that they would stay in Germany, and they would have had to learn. In their daily lives in their communities, they mostly speak their mother tongue and, therefore, German is not needed. However, as the children got older, they suddenly saw the need as they had to stay in Germany and look for a job or task. Participation in the PA programs of the project and *Photovoice* was therefore also seen as an opportunity to speak German and improve their language skills.

We talk about what we have done and how we learn many new words. Because we also still need the language, articles, and words. Then we learn something from each other and culturally, and we ask, "Where are you from?" (Walking to Running, Woman B).

#### 3.1.2. Interpersonal component

Over the course of the project, the women developed an understanding of the social support needed to participate regularly in PA (**critical awareness**).

In this context, most of the women stated that they could not motivate themselves to exercise independently: they needed **the social support of the group or exercise instructors** to attend courses weekly. During the project, they developed the confidence to exercise regularly. Still, the settings (e.g., women-only courses, safe space), the other women, the researchers, and the exercise instructors helped them stay in the programs. Self-directed WhatsApp groups and regular prompts also supported this participation in training. At the same time, a group dynamic and a sense of togetherness were created in the group. Different rules for participation, such as regular attendance and punctuality, were negotiated together.

And when I have time, I only do it in the morning; it’s no fun alone. I always need someone to be there. I'm that kind of person. I always have to have someone to join in, and that's very rare. There are so many girlfriends who are really lazy. But, when I say I made börek, everyone is there. But when I say let's do sports, no one is there (Swimming, Woman D).

**Support from their husband or children** was also seen as essential for regular PA participation. The older generation (parents and parents-in-law) seemed more critical of women’s activities, especially sports. However, that also differs depending on the cultural background (e.g., Muslim).

C: My husband and my children always say, "Mom go, go do sports.”

A: Yes, yes, they support. “Swim and go for a walk!" Do everything. Many Turkish women are, unfortunately, under their in-laws and mothers-in-law. And you're not allowed to do that, yes.

C: Today I can't come, today it's very boring. I have to stay at home and sleep a little. And my son says to me, "Mom, please go do sports, please go. Go. It's good; sport is better. Go." Then my sister-in-law comes, and we go together (Pilates, Women A and C).

The women also developed an **understanding of causal agents in the community** and local authorities. They became aware that they had to talk about the project and publicly state their wishes and ideas to be heard. That implies that they were not seen or recognized by the city council and other city citizens, or they did not feel they were seen and heard.

The exhibition was very nice; it was great, it was something we did. That we talk about the project and what we have done. And our voices were also heard, so lovely. And there are things like that; many women don't have opportunities. But now there is the project, more options, and many women motivate each other (…) and such a voluntary project, that's great (Interview after Exhibition, Woman G).

#### 3.1.3. Behavioral component

Another important aspect of empowerment is networks and the quality of networks. Within the PA programs, the women could connect with exercise instructors and especially women with different migration backgrounds. Most of them have had networks within the same migration background before. Owing to their participation as co-planners and co-researchers, they could also **gain additional social contacts and networks** by connecting with exercise instructors, university members, politicians, and other involved parties and organizations.

Then I said, then I'll join the project. You get to know the people, you get to know the cultures, and so on. Different cultures. We are Turks, and they are Afghans, how they live, and so on; they already have a culture of their own. And we've been living in Germany for a long time; we’ve been in Germany – I think I came here when I was six, six years old. I went to school here and so on, we grew up here. So we like living here. So in the meantime – I like Turkey too, but Germany too. […] We live in two cultures (Walking to Running, Woman A).

### 3.2. Organizational level

During the project, the women had several organizational participation opportunities: (1) assisting others, (2) taking on independent tasks, and (3) participating in or (4) initiating decision-making processes independently.

#### 3.2.1. Assisting others

Some women **assisted the exercise instructors in the implementation of the PA programs**. As participants in the respective programs, they conducted Corona testing for the other participants during the COVID-19 restrictions. They also kept the attendance lists, encouraged all participants of the programs weekly via WhatsApp to come to training regularly, and helped organize sports equipment for the sports courses for all women, among other tasks.

Furthermore, the women **supported the city project office’s coordinator in recruiting participants for the PA programs**. They used their private networks to develop or expand their networks.

So I like it. Yes, I also have all my friends and circle; I also told them. There is such a project, I said to everyone. For example, my girlfriends, a few girlfriends participate (Pilates, Woman A).

However, the women sometimes disagreed with the approach of the project officials (e.g., concerning the handling and recruitment of participants). They would have **liked to have had more of a say** and advocated, for example, for a stricter approach to absenteeism, as the programs were restricted to 15 participants, and some of them (especially swimming programs) had quite long waiting lists.

He is also nice. But [name of the project coordinator in the city of Augsburg], please cross out if someone is away three times. Many women are waiting on the list. Can you give them (the place)? Many ask me: "Swimming, is there still swimming?" Turkish women, I have learned that Turkish women love water (Pilates, Woman A).

Unfortunately, contrary to the women’s wishes, no additional project office position, filled by a woman from the target group as a mini job, was created in the city’s Health Department.

The women likewise **supported the researchers in the implementation of the photo exhibition**. They painted the wall of the exhibition room, decorated the shop windows, designed, and decorated the feedback corner, put the pictures on the walls, and some brought food and Turkish tea for the opening ceremony. Furthermore, they made videos about the exhibition and posted these on Instagram and Facebook. Others helped during the opening hours as supervision and guide, helped clean the exhibition rooms, or wrote quotes of the interviews on the walls.

#### 3.2.2. Taking on independent tasks

After being participants in some programs of the project, two women took on additional **roles as exercise instructors** in the course “Walking to Running” and one in the course “Pilates.” The researcher asked all women face-to-face if they were interested in doing PA courses by themselves. Those women who were interested met with the researcher and an employee from the Bavarian State Sports Association (BLSV, Bayerischer Landessportverband e.V.), responsible for the program “Integration through Sports,” in an extra meeting. There the women were informed about insurance issues, the possibility of acquiring an exercise instructor license by the BSLV, and how to do training and especially encouraged to do so. Twelve women came to the meeting, five were more interested, and so far, three women are teaching PA courses. In addition, 12 women attended a two-hour training course carried out by the city council, which trained them on how to teach, design, and create exercise courses.

And on Sundays, I run with the women. I also talked to [name] yesterday that I should keep doing this (Interview after Exhibition, Woman A).

As part of the *Photovoice* study, the women were asked to document their daily lives in terms of PA and sports. By taking and discussing photos, they **took on the role of co-researchers**. In the focus group discussions, they interpreted their pictures together and explored different topics without being asked about them by the interviewer. As some showed photos of PA and others of doing housework, cooking, of their children and grandchildren, and meeting with friends, they reflected independently on these issues. They discussed them as possible barriers to their participation in PA in the group.

#### 3.2.3. Participating in decision-making processes

At the beginning of the project, several women participated in decision-making processes during **project planning group sessions** (see Methods section). The four different kinds of PA courses realized in project implementation (Moving in the Water, Walking to Running, Pilates, and Self-defense/FitBo courses) were based on the women’s wishes. The following framework conditions highlighted as significant by the women in the cooperative planning process were also implemented: childcare availability, close to home, without costs, and only for females (safe space).

As the project progressed, the women were also **involved in decisions about program content**. For example, as they indicated that “Fit-Bo” was too strenuous, it was replaced with yoga and dancing in consultation with them. The participating Muslim women wanted swimming lessons and expressed pleasure at receiving them through the project. Therefore, in the last block of the PA courses, there were three swimming courses initiated by the women and the researchers.

#### 3.2.4. Initiating decision-making processes on their own

One participant **created a list of possible women interested (150) in swimming lessons** (like an unofficial petition). She showed this list to the researcher. These efforts provided the basis for implementing swimming lessons for women as part of the project. This woman gained insights into different bureaucratic processes and structures associated with implementing PA programs.

When you sign up for it, it has to be binding, and I don't know how you can force people to go ahead or continue. I have now given [name] the list of women; he said there are many women. And if you do all the work and effort, you can see all this bureaucratic stuff, phoning, begging, pleading, and calling everybody, and then they don't come. Either it's too easy for them. They don’t know how much dedication is behind it and how much time and energy (Interview after Exhibition, Woman A).

In the interviews, the women expressed how important the project was for them and wanted it to continue. They used the exhibition with photos and quotes **to describe their needs and wishes to the city officials and ensure the project’s sustainability**. They activated their networks to get as many women as possible to participate and to give their wishes as much emphasis as possible.

That the project always goes on, with swimming, sports, and running. That we always have programs in which we can participate. Many want to do that, but some can't express themselves (Walking to Running, Woman A).

The women were excited but, at the same time, proud to be able to present themselves and their circumstances.

No, it was already good; it was a shaking up that we could say that we were also here. We are invisible in the city because we are not loud, but we are there. Society is like that; you can't just look away. You can’t ignore the refugees or the Ukrainians, or, I don't know, foreigners; they are just there in the city. You can only do something together. Side by side, it would only work for a short time. There would be big problems. And you can only be noncommittal in such groups, get to know each other, and accept and respect the others (Interview after Exhibition, Woman A).

In addition, some face-to-face conversations between stakeholders and women occurred during the exhibition, and the women felt heard and recognized.

The exhibition was excellent for me. Because there was such an exchange, where you saw people from different courses, I say. Because there were people from the university, from the chair, and then politicians and women who were disadvantaged and the target group, there was an exchange. So I was a project participant, a student, and I saw politicians. For example, I saw women from the same environment as me; yes, I saw very different people and talked to them. And there was an intercultural exchange. I liked that very much. *Interviewer*: And did you also talk to politicians and everyone? *G:* Yes, I talked to the artist in charge of the exhibition. I talked to them; I’ve forgotten the name now, but the head of the department (of the university), I talked to him. With the swim class instructor, [Name], with her I spoke, also with someone from the city, so really with everyone (Interview after Exhibition, Woman C).

### 3.3. Community level

#### 3.3.1. Access to communal resources

As most of the participating women are Muslim, their religious background does not allow them to go swimming together with men (or at least they have to wear full-body swimsuits, which are not allowed in every swimming pool of the city of Augsburg, or the women are verbally attacked because of this and get mean looks). The headscarf was likewise perceived as an inhibiting factor for participation in regular PA by the women themselves. The same applies to some of them regarding gender-mixed sports groups or opportunities. When they participated in PA programs with the headscarf, they felt different but did not dare to take it off. At the same time, they felt they did not belong and therefore stopped participating again. Sports facilities for women only, such as women’s fitness centers or other women-only sports groups, were often too far away or too expensive. Furthermore, they had no access to public indoor swimming pools in Augsburg and could not go swimming, as women-only swimming hours were unavailable. However, they were aware that other cities have such offerings.

I wanted to go to the gym once, but it was mixed. And because I wear a headscarf and have to undress if it's too warm, and because it's diverse, I didn't dare, to be honest. So I said, "No" (Walking to Running, Woman A).

Well, it bothers me. Where I was with and without a headscarf, I noticed how people looked at me and treated me. So the person without the headscarf always has priority. I saw that, unfortunately. They also treat you differently; you can feel that. You can think that, you notice that. If you didn't have a headscarf before and how you were treated there and with a headscarf, you see that, the fact that people react very differently. I don't know, with me (Walking to Running, Woman A).

During the project, the women **gained access to women-only indoor pool hours and women-only PA programs** in their neighborhood in the city of Augsburg, organized by the city council, the university, and the insurance companies. They were also given access to a suitable space in the city to exhibit their photos. These concessions not only allowed them to engage in regular PA but also to have a sense of belonging and being heard.

#### 3.3.2. Sense of community

They also got to know other women in quite similar situations through the project. They reciprocally enhanced their understanding of their situation, experienced **cultural openness, and developed an understanding of and with other women** in comparable life situations.

This project is quite good; now we know each other. This generation may open the doors, but our children come together later. Whether the headscarf or other people’s hair is different or dressed differently, this is the opposite side; we get to know them (Walking to Running, Woman B).

In each group interview, at least one woman **stood up for the other women** who could have spoken German better or were introverted.

You know, with many women, it's also the language. They don't dare. Because they don't know German so well, they can't defend themselves (…). Because if someone comes and says something that they don't understand or can’t protect themselves from, they get sad. Then they will not go there anymore. (Pilates, Woman A).

Many Turkish women think they are responsible for the kitchen, husband, cooking [Woman C: cleaning], or cleaning. "No, I can't come; I don't have time for that" (Pilates, Woman A).

#### 3.3.3. Open government structures

The participating women **became politically active** by agreeing to present their photos and interview quotes at the exhibition. Some of them also decided to participate in an additional video shown at the photo exhibition. The video featured short (1–4 min) sequences of participating women talking about who they are and what the project means to them. Two women also took on the role of **speaking at specific project events (opening ceremony of the exhibition, closing ceremony of the project) in front of various stakeholders, such as local policymakers and other relevant authorities** in the city of Augsburg, to inform them about the project and its significance. Here the researchers again asked all women participating in the PA courses to do so.

B: I was excited [researcher's name] because I had nothing prepared.

A: But you spoke like a waterfall.

B: Oh, what, there I have so much grammar.

A: I asked her if she had studied it before. She said, "No."

B: I didn't know either; what must I talk about? Later I said, "Oh, [own name], you talked wrong and didn't say that. But at least we are there. Whether there is something wrong or right." (…) There I had much joy when I participated. That's quite good; no one heard and understood us (before). (Interview after Exhibition, Women A and B).

However, the inclusion of women in the project presentation at various city council committee meetings was rejected several times by those responsible.

## 4. Discussion

### 4.1. Aim of the study

This paper aimed to explore the perspectives of women in difficult life situations on their participation in CBPR projects in terms of empowerment. Following the co-creation approach to health promotion (9), the women were included as co-researchers in the current CBPR project. A *Photovoice* approach was used for this purpose, including focus group discussions with the women and an exhibition of their photos, interview quotes, and short interviews. The study adds value to existing literature on the perceived empowerment effects of women in difficult life situations concerning different modes of participation (43, 44).

### 4.2. Principal findings and comparison with other studies

The principal findings are that the women in the present context perceived effects and processes on all three empowerment levels (i.e., individual, organizational, and community) through their participation in the CBPR project.

As a result of their *participation in the project’s PA programs*, women reported empowerment effects on the individual level: They perceived more self-efficacy regarding regular PA, more control over certain important areas of their life like pain issues, overcoming fatigue, or fear of water, and gained different competencies. The latter point was in terms of PA participation and concerning further relevant areas, such as improving nutritional and German language skills. They also developed an understanding of supportive factors that are significant for them to engage in regular PA: support of family members and friends, as well as the support of a group and the exercise instructor. Furthermore, they made additional social contacts with women, especially from other cultures, due to their participation in the project’s programs. The group acted as a motivator for regular PA participation. This is like findings from other studies on the empowerment effects of women’s participation in PA programs (43, 44, 51).

*Through participation as co-researcher*, the women perceived a set of more generic competencies that impacted their everyday life and enabled them—in terms of the WHO (1997)—to take control of the determinants of their own and other people’s health.

The photo assignment, for example, encouraged the women to reflect together on their daily lives concerning PA and sports. As some women had photos of sports and PA and others had pictures of their household, food, children, and grandchildren, differences in assessing the importance of specific domains became apparent. Finally, in focus groups, the photos served as stimuli to discuss their role as women in the context of the family and other areas of life as well as related possible barriers to their participation in sports and PA. As described by Freire (20), who proposes that photos enable people to reflect on themselves and their world and achieve critical consciousness about their situation, the images serve as a mirror for the participating women (49). Similar effects on raising awareness of the barriers to PA participation through discussing photos in the context of *Photovoice* have been described in previous studies (52). Through the mutual exchange, the women encouraged one another concerning their photos. Finally, they all agreed to show their photos at the exhibition to share their views and lives with policymakers, the media, family, friends, and other interested citizens.

Participating in the focus group discussions, as well as in conversations and as speakers at the exhibition, helped the women improve their German language skills further. This was not only a goal strived for by the women themselves with their participation in the current CBPR project, rather, they regarded language competence as an essential prerequisite for acquiring health literacy (53). In the focus groups, the women learned to better understand different cultural backgrounds and individual desires and needs related to PA. This also contributed to a better sense of community and can be seen as an advantage of group discussions versus individual interviews (54).

*By exhibiting their photos, quotes, and a video*, the women expressed their wishes and needs to relevant city leaders and citizens. With this, the women learned to be capable of influencing and even initiating far-reaching decisions and were valued for their opinion. Some women even gained the confidence to speak and represent the concerns of their peer group in conversations with and in front of city officials and leaders. As reported elsewhere, they felt heard and valued for their beliefs and ideas (55). Thus, they perceived some form of openness of the local government, even though the inclusion of women in the presentation of the project in various committee meetings of the city and their participation as co-project office managers in the city council were rejected several times by those responsible. It appears that the latter changes require patience and time and maybe longer project durations (37). Compared to the experiences from other CBPR projects, the lack of openness of the local government concerning the women’s participation seems quite striking and possibly indicates a lack of readiness for the topic in the present municipality (43, 55, 56). Despite these circumstances, the fact that the project is sustainable is more than remarkable. From our point of view as participant observers, this is due to the exhibition of the photos and the attention the women attracted to their wishes and needs, especially among opposition politicians and the local media. Local newspapers and local social media were specially invited by the researchers (for several reasons). However, the dissemination of the results of *Photovoice* and how exhibitions may influence societal, organizational, and individual changes has yet to be studied and discussed in the literature (49).

*Compared to the involvement of the women as co-planners and co-implementers* (43), quite similar empowerment processes at the organizational and community levels were achieved by their participation as co-researchers through *Photovoice*. By participating as project office managers in a CPBR project for PA promotion (co-implementation), a few women reported developing skills related to office work (43, 44). In addition, the establishment of permanent groups like cooperative planning groups or health promotion labs possibly allows for more intensive and long-term collaboration among target group participants and other stakeholders (43, 54). While the empowerment effects just mentioned could not be shown in the current project, several other benefits of *Photovoice* in terms of empowering target groups in the context of CBPR became apparent. As discussed above, photos could at least partially counteract language problems and enable the inclusion of women who hardly spoke German (22). Additionally, the focus group consisted only of participants from the target group. As there was a relationship of trust between the group participants, they could express themselves much more freely (57). In contrast, a lack of willingness on the part of target groups to participate in mixed stakeholder groups has been described several times in the literature. This is attributed to the fact that, partly due to language problems, they feel uncomfortable in groups with policymakers and are unwilling to speak up (58). The willingness to get involved in informal groups (e.g., at women’s regular breakfasts) is estimated to be significantly higher (44). The basic structure of *Photovoice* allows for a more informal nature of participation. In the present project, the women could and had to decide at any time if they wanted to participate, drop out, or begin. There were no regulations regarding this point. That again is an essential tool to gain empowerment and be responsible for oneself. Quite a few women made their own decision in this regard as well. This may be why the women’s commitment to join and support the project was relatively high.

### 4.3. Strengths and limitations

Our data demonstrate that the women perceived *comprehensive empowerment effects* in terms of self-efficacy and skill development and even gained *empowering processes* in the form of social and community involvement and power through their participation in a CBPR project for PA promotion. In addition, it was shown that the participation of women in difficult life situations in a CBPR program in the form of *Photovoice* could achieve comparable effects on their empowerment as other forms of participation, such as cooperative planning. *Photovoice* can be carried out in different ways. Usually, there is the same focus group of 5–15 persons who meet several times to take pictures, give their photo headings, discuss the photos, and so on. In this project, the women could, at any stage, drop in or out and begin with the co-researching process. It was mostly the same women, but new women also participated in the exhibition preparation.

As reported in other publications, the *concept of empowerment is quite uncertain and fuzzy* (59). Subcategories differentiated by Zimmermann (39) often overlap and, in some cases, can hardly be distinguished. Empowerment is a process that takes time and develops in small steps, and the individual levels are mutually dependent. The willingness to open oneself to different issues, possible change, and modification in life is necessary to gain and learn new skills. Furthermore, skills and readiness are interdependent.

The *role of the researchers* in the present context must also be highlighted: The researchers were very open, flexible, and actively participated in the PA courses throughout the whole CBPR project, resulting in a high level of trust from the participants and an intensive exchange of ideas and needs throughout the whole research project. The times and locations of the focus group discussions were also tailored to the wishes and needs of the women. They mostly took place directly after the PA courses to make it easy for the women to participate. Given the use of WhatsApp groups for all courses, the women often called the researcher quietly when there were problems, new participants, and other concerns. Without this platform, the intensive participation of women in the project would not have been possible. This is critical in going native but is also an essential prerequisite for the participatory, and thus empowering, processes (49). Regarding the participation of the women in the research process, it should be noted that they were involved in data collection, analysis, and dissemination but not in the formulation of the research question. Possibly greater empowerment would have been achieved through an even broader participation of the women (57).

The *group discussions occurred in German* because the women were of different origins (Persian, Turkish, German, and Hebrew). Therefore, most did not use their mother tongue and could not express themselves easily. However, through the photos, these women also had their say, even if some admitted that they did not understand the photo task. As explained above, all the photos were discussed, interpreted, and contextualized in the group. In some interviews, the women helped one another and translated for those who could not speak German well, even though they had lived in Germany for more than 15 years. It should likewise be recognized that the researcher conducting the interviews is a German woman aged 40, and thus power relations could not be excluded.

Since at least one of the two researchers who performed the data analysis had *experience with the research question from previous projects, data analysis in the form of grounded theory* was chosen to be applied to the research object as openly as possible and to focus on how the women perceived their reality. The results and impressions from the analysis of the interviews together with the women were considered several times throughout the process of data analysis. In addition, as already requested by others (35), triangulation of the data and, therefore, different perspectives was carried out by combining interviews with participant observation data.

As the *Photovoice* group interviews took place after a one-year Corona break*, only some of the women involved in cooperative planning participated in Photovoice* because many had dropped out of the project due to changes in their life circumstances (new job, older children, etc.). Therefore, we did not have data on how the women felt about participating in cooperative planning.

### 4.4. Implications for further research and practice

Our findings support the notion that health promotion interventions with marginalized groups can contribute to their empowerment on multiple levels when participants become equal partners in the CBPR project. Including women as research partners in data collection and analysis leads to further empowerment outcomes and has advantages over other ways of participation. However, the inclusion of target groups in all phases of the research project possibly causes a summation and interdependence of effects, an outcome that should be reviewed. For example, to counteract the language problems of participants about understanding the photo assignment, intensive training in *Photovoice* is urgently required beforehand. Family support is essential for women’s participation in PA and sports, so future projects should consider including family members in CBPR. The present study underlines that participation in PA alone already produces empowerment effects at the individual level. This result once again underscores the importance of PA interventions for health promotion.

## Data availability statement

The raw data supporting the conclusions of this article is available upon reasonable request from the corresponding authors.

## Ethics statement

The studies involving human participants were reviewed and approved by Ethik Kommission der Universität Augsburg. The patients/participants provided their written informed consent to participate in this study.

## Author contributions

UR-O and HB-B initiated the study. UR-O designed the study, analyzed the data, and drafted the manuscript. EK conducted Photovoice, analyzed the data, and contributed to the draft of the manuscript. HB-B contributed to the study conception and critically revised the manuscript. All authors read and approved the final manuscript.

## Funding

The described project was funded by the community of health insurance companies in Bavaria. The open access publication of this article was supported by the DFG sponsored Open Access Fund of the University of Augsburg.

## Conflict of interest

The authors declare that the research was conducted without any commercial or financial relationships that could be construed as a potential conflict of interest.

## Publisher’s note

All claims expressed in this article are solely those of the authors and do not necessarily represent those of their affiliated organizations, or those of the publisher, the editors and the reviewers. Any product that may be evaluated in this article, or claim that may be made by its manufacturer, is not guaranteed or endorsed by the publisher.

## Footnotes

^1^According to the BIG project, a project for the promotion of PA among women in difficult life situations, these women were defined as those who are unemployed, have low income or receive social welfare, have low educational attainment, have a migration background, or are single mothers (37).

^2^Session 1: Getting to know each other and Brainstorming, Session 2: Prioritization, Sessions 3–6: Action planning, Session 7: Adoption of a catalog of measures.
